# Mycobacterial Epoxide Hydrolase EphD Is Inhibited by Urea and Thiourea Derivatives

**DOI:** 10.3390/ijms22062884

**Published:** 2021-03-12

**Authors:** Jan Madacki, Martin Kopál, Mary Jackson, Jana Korduláková

**Affiliations:** 1 Department of Biochemistry, Faculty of Natural Sciences, Comenius University in Bratislava, Mlynská Dolina, Ilkovičova 6, 842 15 Bratislava, Slovakia; jan.madacki@gmail.com (J.M.); rndr.martin.kopal@gmail.com (M.K.); 2 Mycobacteria Research Laboratories, Department of Microbiology, Immunology and Pathology, Colorado State University, Fort Collins, CO 80523-1682, USA; mary.jackson@colostate.edu

**Keywords:** mycobacterium, epoxide hydrolase, isoxyl, thiacetazone, AU1235, mycolic acids

## Abstract

The genome of the human intracellular pathogen *Mycobacterium tuberculosis* encodes an unusually large number of epoxide hydrolases, which are thought to be involved in lipid metabolism and detoxification reactions needed to endure the hostile environment of host macrophages. These enzymes therefore represent suitable targets for compounds such as urea derivatives, which are known inhibitors of soluble epoxide hydrolases. In this work, we studied in vitro the effect of the thiourea drug isoxyl on six epoxide hydrolases of *M. tuberculosis* using a fatty acid substrate. We show that one of the proteins inhibited by isoxyl is EphD, an enzyme involved in the metabolism of mycolic acids, key components of the mycobacterial cell wall. By analyzing mycolic acid profiles, we demonstrate the inhibition of EphD epoxide hydrolase activity by isoxyl and two other urea-based inhibitors, thiacetazone and AU1235, inside the mycobacterial cell.

## 1. Introduction

With about 10 million new cases and 1.4 million deaths in 2019, tuberculosis (TB), a disease caused by pathogenic bacterium *Mycobacterium tuberculosis*, remains a serious global health problem. The End TB Strategy adopted by the World Health Organization (WHO) envisioned a 20% decline in global incidence of TB and a 35% reduction in TB deaths between 2015 and 2020; however, despite some progress, these milestones have not been reached [1]. Accelerating this decline and putting a stop to the TB epidemic requires intensifying the search for a new vaccine and new chemotherapeutics, tasks which face several challenges [2,3,4]. Currently, a six-month regimen is routinely implemented in treatment of drug susceptible TB, consisting of a combination of isoniazid (INH), rifampicin (RIF), ethambutol (EMB) and pyrazinamide (PZA). In the case of multiple-drug-resistant TB (MDR-TB), the treatment can last up to 20 months and involves administration of second-line TB drugs, which are associated with more severe side effects and toxicity [5].

**Figure 1:**
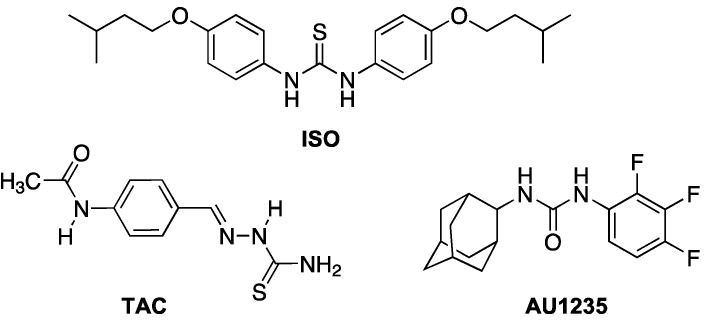
Chemical structures of isoxyl (ISO), thiacetazone (TAC) and AU1235.

Isoxyl (thiocarlide; 4,4′-diisoamyloxydiphenylthiourea) (ISO) (Figure 1) is a thiourea compound that was used for treatment of TB in the 1960s, but its use was discontinued due to poor bioavailability [6]. Nevertheless, ISO shows potent antimycobacterial activity in vitro with low minimal inhibitory concentrations (MICs) on *M. tuberculosis,* including strains resistant to RIF and INH [7]. Initial studies found that ISO inhibits the stearoyl-CoA Δ9-desaturase DesA3. However, this did not explain the inhibition of mycolic acids seen in ISO treated *M. tuberculosis* [8]. Mycolic acids are very long chain (C60–C90) α-alkyl β-hydroxy fatty acids and are key components of the mycobacterial outer membrane (mycomembrane), their presence being essential for the viability of the bacterium [9,10]. Mycobacteria produce several types of mycolates differing in the presence of different functional groups in their hydrocarbon chain, such as double bonds, cyclopropane rings and oxygenated functions [11]. In subsequent studies, it was shown that ISO and thiacetazone (TAC), a related thiosemicarbazone drug (Figure 1), both target HadAB, the β-hydroxyacyl-ACP dehydratase component of the fatty acid synthase II (FASII) system, which is responsible for the synthesis of mycolic acids [12]. It is well established that ISO and TAC are prodrugs and require modification by the mycobacterial monooxygenase EthA for activity [13,14,15].

Mycobacteria have an extremely hydrophobic cell envelope that consists of a conventional plasma membrane; a layer of peptidoglycan; and a polysaccharide, arabinogalactan, which is esterified by mycolic acids, forming the inner leaflet of the mycomembrane [16]. The outer leaflet of the mycomembrane is composed of various complex lipids such as trehalose dimycolate (TDM), di- and polyacyl trehaloses, phthiocerol dimycocerosates and sulfolipids [17,18]. The mycobacterial cell envelope is an important target for antimycobacterial compounds, with the most notable examples being first-line antituberculotics INH, which inhibits the FASII enoyl-ACP reductase InhA [19,20], and EMB, which interferes with the synthesis of arabinogalactan [21]. In recent years, several new inhibitors were found to target the cell envelope, for example, by affecting the transporter MmpL3, which transfers trehalose monomycolate (TMM) across the plasma membrane [22]. Once transferred to the periplasmic space, TMM is a substrate for the mycolyltransferases FbpA, FbpB and FbpC (also known collectively as the antigen 85 complex) that catalyze the transfer of mycolic acyl chains onto cell wall arabinogalactan or the synthesis of trehalose dimycolate (TDM), thus incorporating mycolates into the mycomembrane [23]. One of the MmpL3 inhibitors is an adamantyl urea AU1235 (Figure 1), which inhibits a range of mycobacterial strains, including MDR isolates of *M. tuberculosis* [22].

Initial analysis of the genome of virulent strain *M. tuberculosis* H37Rv discovered five genes encoding putative epoxide hydrolases (EHs) homologous to eukaryotic α/β hydrolase fold EHs, each about 300 amino acids long (*ephA, ephB, ephC, ephE* and *ephF*), and another one which appeared to be an N-terminal part of a fusion protein with a short-chain dehydrogenase (SDR) at its C-terminus, named *ephD* [24,25]. Another EH from *M. tuberculosis* (EphG) homologous to the limonene 1,2-epoxide hydrolase from *Rhodococcus erythropolis* was described [26]. Recently, we were able to produce these seven mycobacterial EHs in soluble form and we have shown, by using 9,10-*cis*-epoxystearic acid as substrate, that, with the exception of EphC, all of them display epoxide hydrolase activity in vitro. At the same time, we demonstrated that the N-terminal EH domain of EphD is responsible for its EH activity, which, in turn, results in the ability of this protein to modify epoxymycolates in non-pathogenic *Mycobacterium smegmatis* [27]. To date, structures of several EHs from *M. tuberculosis* or their orthologues from *Mycobacterium thermoresistibile* were solved [26,28,29], and although their physiological substrates still remain to be discovered, it was proposed that they might be important in detoxification reactions [25].

It is known that human-soluble epoxide hydrolase (sEH) can be inhibited by different urea derivatives [30,31], which was also shown for mycobacterial EHs EphB and EphE [32,33]. In this work we tested the activity of EphA, EphB, EphD, EphE, EphF and EphG in vitro on 9,10-*cis*-epoxystearic acid in the presence of ISO. We show that the activity of EphD is inhibited inside mycobacterial cells by ISO, TAC and AU1235, and that this enzyme is thus an additional target for these antimycobacterial compounds.

## 2. Results

### 2.1. Monitoring the Activity of Mycobacterial EHs in the Presence of ISO

**Figure 2:**
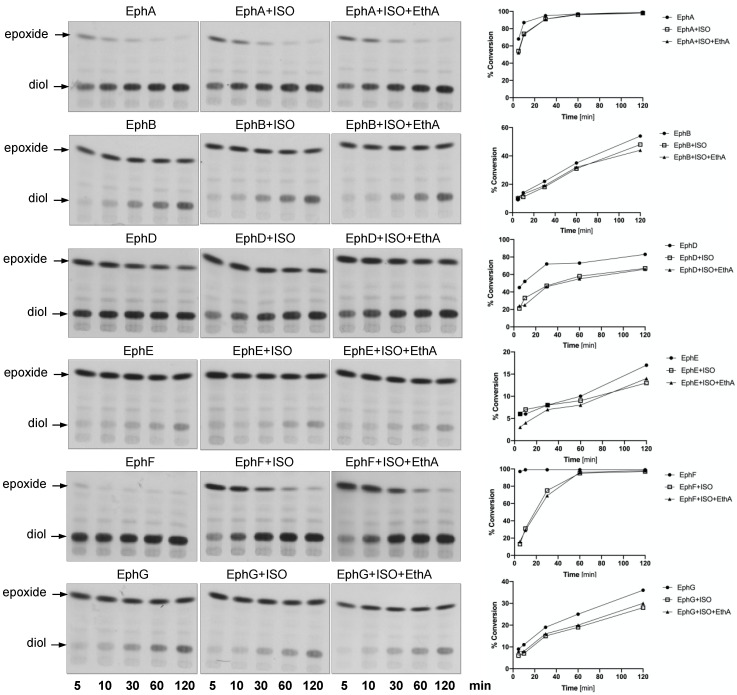
Epoxide hydrolase activity of EphA, EphB, EphD, EphE, EphF and EphG proteins in vitro in the presence of 10 µg/mL of ISO. Left: Thin-layer chromatography of in vitro reactions with 10,000× *g* supernatants of lysates of *E. coli* strains producing EHs from *M. tuberculosis* using [^14^C]-9,10-*cis*-epoxystearic acid as the substrate. Reactions ran for 5, 10, 30, 60 or 120 min at 37 °C. Plates were developed in *n*-hexane:diethyl ether:formic acid (70:30:2) and visualized by autoradiography. Right: Quantification of results of thin-layer chromatography (TLC) analysis. For each condition, the EH activity was expressed as percentage of substrate conversion.

In order to monitor the EH activity of mycobacterial EHs in vitro, we used a radioactively labelled substrate, [^14^C]-9,10-*cis*-epoxystearic acid [34,35] and 10,000× *g* supernatants of the lysates of *E. coli* strains individually producing each EH—*E. coli* BL21(DE3) pET29a-*ephA*, *E. coli* BL21-AI pDEST17-*ephB*, *E. coli* BL21(DE3) pET29a-*ephD*, *E. coli* BL21-AI pET28a-*ephE*, *E. coli* BL21(DE3) pET28a-*ephF* and *E. coli* BL21(DE3) pET29a-*ephG*—as enzyme sources. ISO was added to the reactions at a final concentration of 10 µg/mL and left incubating for 5, 10, 30, 60 or 120 min. Partially purified monooxygenase EthA was added to some reaction mixtures together with ISO. Extracted lipids were separated by thin-layer chromatography (Figure 2, left), and the bands corresponding to the substrate, [^14^C]-9,10-*cis*-epoxystearic acid or the corresponding diol product were isolated by preparative thin-layer chromatography and quantified by scintillation counting. The EH activity in each reaction was expressed as a substrate conversion percentage (Figure 2, right; Table S1). With the exception of EphD and EphF, the activities of the studied EHs were not significantly affected by ISO. Moreover, in the case of all the EHs tested, we did not observe any significant effect of the addition of EthA to the reactions and, as expected, the proteins of the *E. coli* BL21(DE3) pET28a lysate did not catalyze the conversion of [^14^C]-9,10-*cis*-epoxystearic acid to the corresponding diol (Figure S1).

### 2.2. EphD and EphF Interact Differently with ISO

**Figure 3:**
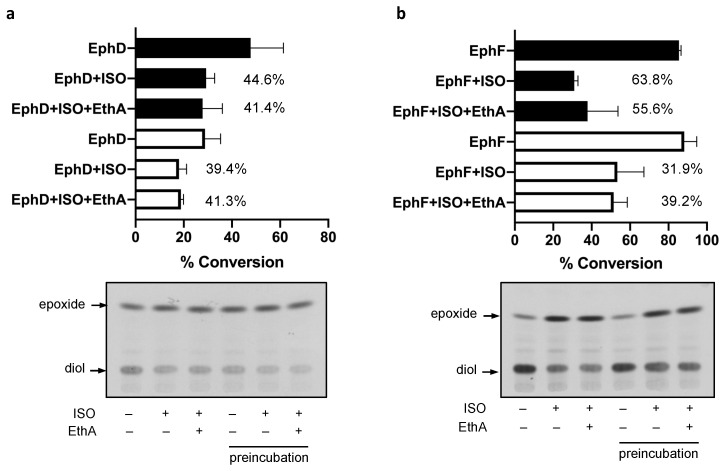
In vitro EH activity of EphD (**a**) and EphF (**b**) in the case of preincubation with 10 µg/mL of isoxyl (thiocarlide; 4,4′-diisoamyloxydiphenylthiourea) (ISO). Bar graph representing average percentages of substrate conversion from two experiments (error bars showing standard deviation). Filled bars represent reactions where ISO (or DMSO in control reactions) was added directly with other reaction components, empty bars represent reactions where the enzyme was incubated with ISO for 15 min prior to adding the substrate. Numbers next to bars represent average percentage of inhibition per reaction. The lower part of the figure shows one representative chromatogram from the TLC analysis of these reaction products. The presence (+) or absence (–) of ISO and EthA is indicated for each reaction.

The activities of EphD and EphF are inhibited by ISO; however, there seemed to be a marked difference in the kinetics of this inhibition—in contrast to EphD, the activity of EphF was inhibited only at the beginning of the incubation and the conversion was comparable to that of the untreated control at later time points (Figure 2). We therefore included a 15 min preincubation step with ISO before adding the substrate and allowing the reaction to run for 15 min. We observed that in the case of EphD, preincubation does not alter the inhibitory effect of ISO, as the percentage of inhibition remains at the same level when compared to the corresponding control reaction (Figure 3a). On the other hand, the preincubation step with EphF resulted in lower inhibitory activity of ISO (Figure 3b), clearly suggesting a different type of interaction between the enzyme and the inhibitor. Again, the presence of monooxygenase EthA in the reaction did not affect the inhibitory activity of ISO.

### 2.3. EphD Is Inhibited by Urea and Thiourea Derivatives inside the Cell

We next sought to determine whether the inhibition of EphD by ISO observed in vitro translated into EphD inhibition inside the bacteria. In our previous work, we showed that protein EphD is involved in the modification of oxygenated mycolic acids in mycobacteria [27]. The overproduction of EphD and its orthologue from *M. smegmatis* mc^2^155, MSMEG_4280, in *M. smegmatis* led to a decrease in the amounts of epoxymycolic acids, which was accompanied by the accumulation of dicarboxymycolates in mycolic acid extracts. Dicarboxymycolates are formed during isolation of mycolic acids and are products of saponification of wax-ester mycolates, which probably arise upon opening of the oxirane ring by EphD. We reasoned that if ISO inhibits EphD inside the mycobacterial cells, cultivation of *M. smegmatis* EphD and MSMEG_4280 overproducing strains in the presence of ISO might lead to a decrease in dicarboxymycolates in these strains. In parallel, we also tested the inhibitory activity of thiosemicarbazone TAC and adamantyl urea AU1235, while INH was used as a control drug.

**Figure 4:**
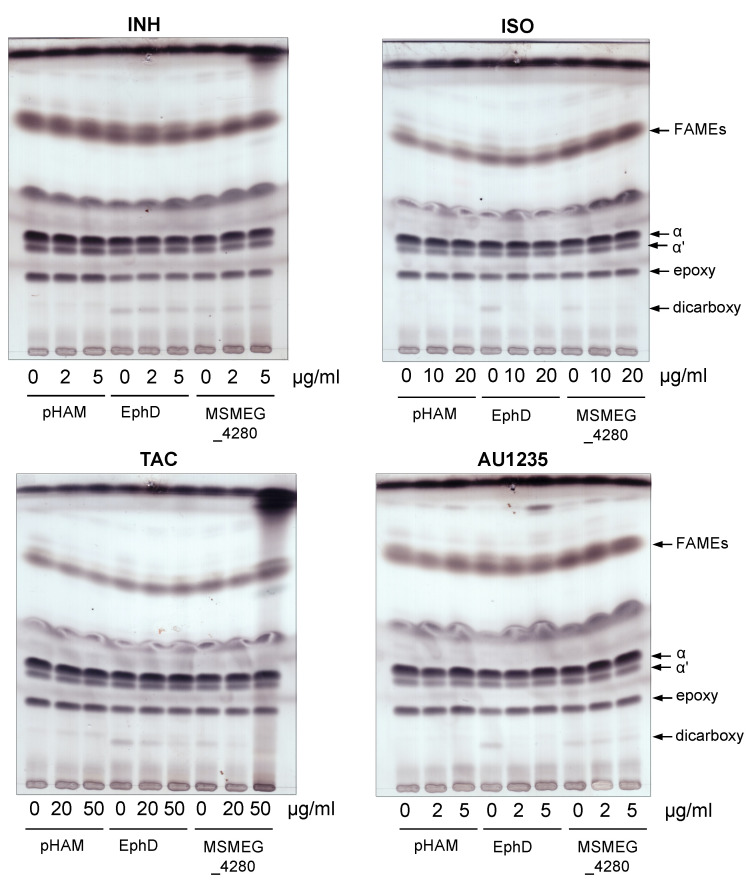
Thin-layer chromatography of fatty and mycolic acid methyl esters from strains *M. smegmatis* mc^2^155 pHAM, pHAM-*ephD* and pHAM-*msmeg_4280* treated with isoniazid (INH), ISO, thiacetazone (TAC) or AU1235. Plates were developed in *n*-hexane:ethyl acetate (95:5, three runs) and visualized with 10% cupric sulfate in 8% phosphoric acid and charring. FAMEs—fatty acid methyl esters; α, αʹ, epoxy and dicarboxy represent different forms of mycolic acid methyl esters (MAMEs).

*M. smegmatis* is intrinsically resistant to ISO and TAC, probably due to the presence of an additional dehydratase, which is able to substitute for the loss of HadAB activity [36]. Higher concentrations of ISO (10 and 20 µg/mL) and TAC (20 and 50 µg/mL) than the MICs of these drugs for *M. tuberculosis* were therefore chosen for our analysis. The concentrations of AU1235 used were lower (2 µg/mL) and higher (5 µg/mL) than the reported MIC of 3.2 µg/mL for *M. smegmatis* [22]. For overproduction of EphD and MSMEG_4280 in *M. smegmatis*, we used an acetamide inducible expression system pHAM [37], and INH, ISO, TAC or AU1235 were added at the same time as the inducer. After 24 h of incubation, the cells were harvested and mycolic acids were extracted and methylated and the resulting mycolic acid methyl esters (MAMEs) were analyzed by thin-layer chromatography (TLC). As observed previously, the overproduction of EphD and MSMEG_4280, compared to control strain, led to lower amounts of epoxymycolates and concomitant accumulation of dicarboxymycolates, while the non-oxygenated α and α’ forms were not affected. In the presence of sub-inhibitory concentrations of INH, the profiles of control strain *M. smegmatis* pHAM as well as overproducing strains remained unchanged (Figure 4, upper left). However, addition of ISO, TAC and AU1235 to the overproducing strains caused a decrease of dicarboxymycolates compared to their untreated controls (Figure 4 and Figure S2), suggesting that the EH activity of EphD is targeted by these drugs inside the cells.

## 3. Discussion

*Mycobacterium tuberculosis* produces an unusually large number of EHs, which probably arose from lateral gene transfer events [38]. As the primary function of this class of enzymes is elimination of toxic epoxides by converting them to less reactive diols, it is tempting to speculate that in *M. tuberculosis* their function lies in suppressing the oxidative damage caused by reactive oxygen species produced by host macrophages. To date, however, data about the native substrates of most mycobacterial EHs are still lacking. Our previous work has shown that EphD, a protein with an N-terminal α/β hydrolase domain and a C-terminal short-chain dehydrogenase domain, is involved in the metabolism of mycolic acids, essential cell-wall lipids of mycobacteria. In the same work we have demonstrated that mycobacterial proteins EphA, EphB, EphD, EphE, EphF and EphG, but not EphC, can cleave 9,10-*cis*-epoxystearic acid into a corresponding diol. In our current study, we used the same substrate to monitor the activities of these EHs in the presence of ISO and we show that, in our experimental setup, only the activities of EphD and EphF are significantly inhibited by this thiourea drug. Brown et al. have observed no inhibition of EH activity by ISO when using purified EphB and a relatively low inhibition when using a crude extract of an *E. coli* strain producing EphE, both on a fluorescent substrate [33]. Our results confirm that ISO does not inhibit EphB, regardless of the enzyme source or substrate used. However, due to low activity of EphE monitored in our assay, we cannot make any conclusion regarding the inhibition of this epoxide hydrolase. Interestingly, while the EH activity of EphD is clearly inhibited by ISO throughout the experiment, in case of EphF, after strong initial inhibition, the substrate conversion returns to control levels over time. This may reflect different binding affinity of the inhibitor, as seen in the case of murine and mammalian sEH [39], or it may indicate a modification of ISO by EphF, which recognizes ISO as a substrate and inactivates this drug. Further efforts are needed to elucidate the interaction between EphF and ISO, the most crucial being the isolation of the enzyme and subsequent structural analysis of possible ISO metabolites produced by catalytic activity of this protein.

Our in vitro assays show that the mycobacterial monooxygenase EthA, which is responsible for the activation of ISO does not affect the inhibition of mycobacterial EHs. It was shown that for exerting their effect on mycobacterial dehydratase HadAB, ISO and TAC need to be metabolized to their sulfenic acid derivatives and as such they covalently bind to the Cys166 residue of HadA, blocking the binding of its physiological substrate [14]. Inhibition of EHs by urea compounds, on the other hand, functions by mimicking the transition state of the substrate upon oxirane ring opening, without prior activation [40,41]. The difference in the mode of action of these inhibitors on the two classes of enzymes might therefore explain that no activation by EthA is needed for the inhibition of mycobacterial EHs.

The clear inhibition of EphD EH activity by ISO in in vitro conditions prompted us to test this inhibition in mycobacterial cells. We took advantage of a direct consequence of the EH activity of this enzyme in the occurrence of dicarboxymycolates in MAME extracts of overproducing strains of *M. smegmatis*. We confirmed that ISO, as well as structurally related drug TAC and adamantyl urea AU1235, inhibit EphD inside the mycobacterial cells, showing that (thio)urea-based inhibitors of different structures can inhibit this enzyme. Although a non-essential gene for in vitro growth of *M. tuberculosis* [27,42], *ephD* is one of the 126 genes shown to be essential for its survival in macrophages [43]. We have observed that deletion of *ephD* in *M. tuberculosis* causes a defect in synthesis of ketomycolates, which appear to be important for TB pathogenesis [44,45]. However, it remains to be determined what is the precise role of EphD in the biosynthesis of this mycolate class and how exactly are its EH and SDR domains involved in this process.

Altogether, our results support the notion that an approach consisting of refurbishing known drugs with strong antimycobacterial activity, such as urea and thiourea derivatives, and improving their pharmacokinetic properties is worth considering in combating *M. tuberculosis*. Several works [33,46,47,48] indicate that structural optimization of these compounds might indeed lead to promising results.

## 4. Materials and Methods

### 4.1. Cloning, Bacterial Strains, Growth Conditions and Recombinant Protein Production

For cloning purposes, strain *E. coli* DH5α (Stratagene, San Diego, CA, USA) was used and cells were grown in Luria-Bertani (LB) medium (Invitrogen, Carlsbad, CA, USA) with shaking or on LB agar plates at 37 °C. The coding sequence of gene *ethA* was excised from the construct pVV16-*ethA* [15] using restriction endonucleases *Nde*I and *Hind*III and cloned into pET29a(+) (Novagen, Piacenza, Italy). The resulting construct pET29a-*ethA* was transformed into *E. coli* BL21(DE3) (Stratagene).

Strains *E. coli* BL21(DE3) pET29a-*ephA*, *E. coli* BL21-AI pDEST17-*ephB*, *E. coli* BL21(DE3) pET29a-*ephD*, *E. coli* BL21-AI pET28a-*ephE*, *E. coli* BL21(DE3) pET28a-*ephF*, *E. coli* BL21(DE3) pET29a-*ephG* [27] and *E. coli* BL21(DE3) pET29a-*ethA* were grown in Luria-Bertani (LB) medium (Invitrogen) at 37 °C with shaking, until optical density at 600 nm (OD_600_) reached 0.4–0.6, when recombinant protein production was induced by adding 0.4 mM isopropyl-β-D-1-thiogalactopyranoside. Strains were further incubated for 16 h at 16 °C, except for *E. coli* BL21(DE3) pET29a-*ephD,* which was incubated at 30 °C for 4 h.

Strains *M. smegmatis* mc^2^155 pHAM, *M. smegmatis* mc^2^155 pHAM-*ephD* and pHAM-*msmeg_4280* [27] were grown in liquid modified M63 medium (7.6 × 10^−2^ M (NH_4_)_2_SO_4_, 0.5 M KH_2_PO_4_, 1 mM MgSO_4_, 0.2% succinate, 0.025% tyloxapol, pH 7) at 30 °C with shaking, to an OD_600_ of 0.6–0.9, when acetamide was added to a concentration of 0.2%. At the same time, INH (0, 2 and 5 µg/mL), ISO (0, 10 and 20 µg/mL), TAC (0, 20 and 50 µg/mL) or AU1235 (0, 2 and 5 µg/mL) were added with the final concentration of dimethyl sulfoxide (DMSO) of 1% and cultures were further grown for 24 h at 30 °C.

When needed, 20 µg/mL hygromycin and 20 µg/mL kanamycin were added to the medium.

### 4.2. Purification of EthA

The recombinant His-tagged protein EthA was isolated from *E. coli* BL21(DE3) pET29a-*ethA.* One gram (wet weight) of cells was resuspended in 4 mL of buffer A (25 mM Tris-HCl, pH = 7.5, 10% glycerol, 300 mM NaCl) with addition of DNase (1.5 µg/mL). The suspension was subjected to probe sonication (10 times 20 s pulses with 60 s cooling intervals), and the sonicate was centrifuged for 10 min, 10,000× *g* at 4 °C. The resulting supernatant was loaded onto 2 mL of TALON metal affinity resin (Clontech, Mountain View, CA, USA), and the unbound protein was removed by washing with buffer A. His-tagged EthA was gradually eluted with buffer A containing 10 mM, 50 mM and 300 mM imidazole, and fractions containing the partially purified protein were combined, desalted using a PD-10 column (GE Healthcare, Chicago, IL, USA) and concentrated using Amicon Ultra-4 (Merck, Kenilworth, NJ, USA) centrifugal filter.

### 4.3. Epoxide Hydrolase Assays

Cell lysates of *E. coli* BL21(DE3) pET29a-*ephA*, *E. coli* BL21-AI pDEST17-*ephB*, *E. coli* BL21(DE3) pET29a-*ephD*, *E. coli* BL21-AI pET28a-*ephE*, *E. coli* BL21(DE3) pET28a-*ephF* and *E. coli* BL21(DE3) pET29a-*ephG* were prepared as described earlier [27], and these were centrifuged for 10 min at 10,000× *g*. EH assays [34] were performed with equivalents of 130 μg of proteins from 10,000× *g* supernatants prepared from the above mentioned strains, with the exception of BL21(DE3) pET29a-*ephA* and BL21-AI pDEST17-*ephB* 10,000× *g* supernatants, where 2 μg and 500 μg of proteins were used, respectively. The reaction mixture contained 22,000 dpm of [^14^C]-9,10-*cis*-epoxystearic acid (56 mCi/mmol) as the substrate, ISO in a final concentration 10 µg/mL (2% DMSO) and, alternatively, 10 μg of partially purified monooxygenase EthA. Adding higher concentrations of ISO was also attempted; however, this caused precipitation of the compound. Reaction volume was adjusted with 50 mM Tris-HCl pH = 7.5 to 250 μL, and the reactions were incubated at 37 °C for 5, 10, 30, 60 or 120 min. For preincubation experiments, all reaction components except the substrate were incubated for 15 min at 37 °C with ISO, after which the substrate was added, and the mixture was incubated for additional 15 min at 37 °C. Reactions were stopped by adding 500 μL of ethyl acetate and vortexing. The samples were further briefly centrifuged, and the upper organic phase was transferred to a clean tube, dried under a stream of nitrogen and resuspended in 20 μL of CHCl_3_:CH_3_OH (2:1). Half of the suspension was analyzed by thin-layer chromatography (TLC) on Silica gel 60 F_254_ plates (Merck) in *n*-hexane:diethyl ether:formic acid (70:30:2), and the radiolabeled compounds were visualized on Kodak BioMax MR film.

For quantification, spots corresponding to the substrate and the product from each reaction were carefully scraped off silica plates, 5 mL of EcoLite(+) liquid scintillation cocktail (MP Biomedicals, Irvine, CA, USA) was added and samples were analyzed on TriCarb 2900TR (PerkinElmer, Waltham, MA, USA).

### 4.4. Analysis of Mycolic Acids

Mycolic acids were isolated as described earlier [7]. Briefly, 1 mL of 15% tetrabutylammonium hydroxide (TBAH) was added to *M. smegmatis* mc^2^155 pHAM, *M. smegmatis* mc^2^155 pHAM-*ephD* and pHAM-*msmeg_4280* cell pellets (0.1–0.5 g wet weight) and the samples were incubated for 100 °C overnight. One milliliter of water, 1.5 mL dichloromethane and 150 µL of iodomethane were then added to the saponified mycolic acids, and they were left shaking for 4 h at room temperature. The organic phase was then washed twice with water and dried under nitrogen flow, and fatty acid and mycolic acid methyl esters (FAMEs, MAMEs) were extracted from the dry residue with 2 mL of diethyl ether. Extracts were transferred to clean tubes, dried, and resuspended in CHCl_3_:CH_3_OH (2:1) according to OD_600_ of cultures at time of harvesting. Samples were analyzed by TLC on Silica gel 60 F_254_ plates (Merck) in *n*-hexane:ethyl acetate (95:5, three runs), and different MAME populations were visualized with 10% cupric sulfate in 8% phosphoric acid and charring.

## Data Availability

Data is contained within the article or Supplementary Material.

## Supplementary Materials

The following are available online at https://www.mdpi.com/1422-0067/22/6/2884/s1. Reference [49] are cited in the supplementary materials.
