# Supplementary material for: Mycobacterial Epoxide Hydrolase EphD Is Inhibited by Urea and Thiourea Derivatives
Source: Int J Mol Sci. 2021 Mar 12;22(6):2884. doi: 10.3390/ijms22062884 (PMC7998700; doi:10.3390/ijms22062884)
Supplement: Supplementary file 1 [file ijms-22-02884-s001.pdf]

Supplementary materials

Mycobacterial Epoxide Hydrolase EphD Is Inhibited by Urea and Thiourea Derivatives

Jan Madacki [1], Martin Kopál [1], Mary Jackson [2] and Jana Korduláková [1,*]

[1] Department of Biochemistry, Comenius University in Bratislava, Faculty of Natural Sciences, Mlynská Dolina, Ilkovičova 6, 842 15 Bratislava, Slovakia; jan.madacki@gmail.com (J.M.); rndr.martin.kopal@gmail.com (M.K.)

[2] Mycobacteria Research Laboratories, Department of Microbiology, Immunology and Pathology, Colorado State University, Fort Collins, CO 80523-1682, USA; mary.jackson@colostate.edu

* Correspondence: jana.kordulakova@uniba.sk

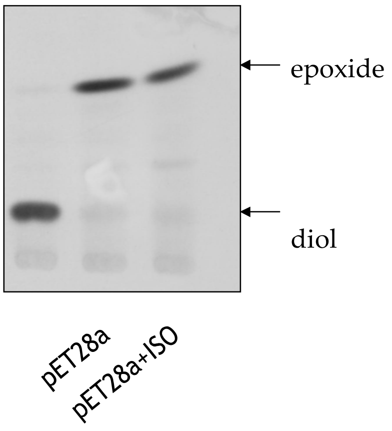

Figure S1. Control of epoxide hydrolase activity in 10 000 × g supernatants of lysates of *E. coli* BL21(DE3) pET28a (an equivalent of 200 µg of proteins) with or without addition of ISO (final concentration of 10 µg/ml). Thin-layer chromatography of *in vitro* reactions using [^{14}C]-9,10-*cis*-epoxystearic acid as substrate. Reactions ran for 30 minutes at 37°C. Plates were developed in *n*-hexane: diethyl ether: formic acid (70: 30: 2) and visualized by autoradiography.

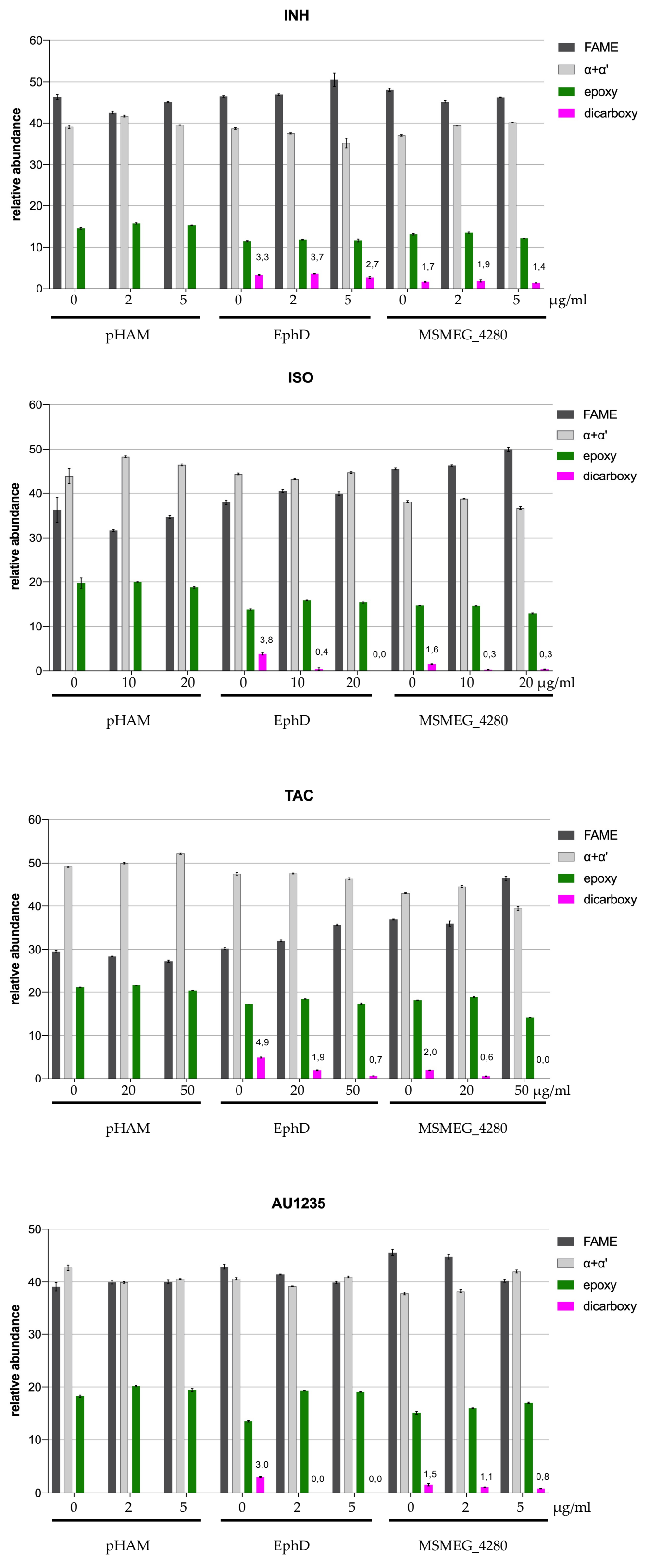

Figure S2. Quantification of fatty and mycolic acid methyl esters from strains *M. smegmatis* mc^{2}155 pHAM, pHAM- *ephD* and pHAM-*msmeg_4280* treated with INH, ISO, TAC or AU1235. ImageJ software (NIH) [49] was used to calculate the relative abundance of fatty acid methyl esters (FAME), α- and α'-mycolic acid methyl esters (α+α'), epoxymycolic acid methyl esters (epoxy) and dicarboxymycolic acid methyl esters (dicarboxy) in each lane from Figure 4. Three independent calculations from the same figure were performed. Bars represent mean ± SD. Individual mean values for dicarboxymycolic acid methyl esters are also written above each corresponding bar.

Table S1. Quantification of substrate and product from each reaction where epoxide hydrolase activity of individual epoxide hydrolases was tested in the presence of isoxyl (10 µg/ml). dpm – disintegrations per minute; Sub. – substrate; Prod. – product; Conv. – substrate conversion.

Time (min)	Sub. (dpm)	Prod. (dpm)	Sub. + Prod.	Conv. (%)	Sub. (dpm)	Prod. (dpm)	Sub. + Prod.	Conv. (%)	Sub. (dpm)	Prod. (dpm)	Sub. + Prod.	Conv. (%)
	pET28a				pET28a+ISO							
30	10720	410	11130	4	5797	223	6020	4				
	EphA				EphA+ISO				EphA+ISO+EthA			
5	1073	2262	3335	68	1749	2033	3782	54	1554	1656	3210	52
10	419	2931	3350	87	981	2838	3819	74	1048	2878	3926	73
30	222	4266	4488	95	334	3207	3541	91	409	3901	4310	91
60	123	4748	4871	97	169	4141	4310	96	160	5396	5556	97
120	70	5257	5327	99	108	4330	4438	98	109	5523	5632	98
	EphB				EphB+ISO				EphB+ISO+EthA			
5	3054	316	3370	9	3664	385	4049	10	2510	305	2815	11
10	4006	663	4669	14	4172	529	4701	11	2855	437	3292	13
30	3696	1069	4765	22	4721	1041	5762	18	3150	743	3893	19
60	2809	1499	4308	35	3522	1608	5130	31	2597	1209	3806	32
120	2464	2908	5372	54	3080	2796	5876	48	2966	2306	5272	44
	EphD				EphD+ISO				EphD+ISO+EthA			
5	2110	1727	3837	45	3746	975	4721	21	3633	1119	4752	24
10	1931	2125	4056	52	2885	1436	4321	33	3383	1142	4525	25
30	1301	3294	4595	72	2306	2083	4389	47	2500	2104	4604	46
60	1051	2898	3949	73	1879	2550	4429	58	2090	2579	4669	55
120	769	3682	4451	83	1609	3208	4817	67	1890	3644	5534	66
	EphE				EphE+ISO				EphE+ISO+EthA			
5	3864	232	4096	6	8938	542	9480	6	4280	155	4435	3
10	4124	269	4393	6	3630	287	3917	7	4543	193	4736	4
30	3879	351	4230	8	3750	311	4061	8	4380	311	4691	7
60	2813	313	3126	10	4308	413	4721	9	4217	368	4585	8
120	3154	639	3793	17	3743	580	4323	13	3561	567	4128	14
	EphF				EphF+ISO				EphF+ISO+EthA			
5	135	3725	3860	97	4050	609	4659	13	3293	615	3908	16
10	40	3018	3058	99	2896	1287	4183	31	2856	1178	4043	29
30	40	4514	4554	99	968	2967	3935	75	1220	2730	3950	69
60	51	4028	4079	99	238	4362	4600	95	169	3937	4106	96
120	41	3654	3695	99	122	4685	4807	97	76	3673	3749	98
	EphG				EphG+ISO				EphG+ISO+EthA			
5	4979	479	5458	9	5124	353	5477	6	2308	205	2513	8
10	5099	608	5707	11	4463	338	4801	7	4159	350	4509	8
30	3726	877	4603	19	4356	759	5115	15	3791	697	4488	16
60	3785	1281	5066	25	3699	843	4542	19	3345	857	4202	20
120	3433	1929	5362	36	3702	1430	5132	28	2973	1269	4242	30
